# Influence of Basalt Aggregate Crushing Technology on Its Geometrical Properties—Preliminary Studies

**DOI:** 10.3390/ma16020602

**Published:** 2023-01-08

**Authors:** Magdalena Duchnowska, Paweł Strzałkowski, Alicja Bakalarz, Urszula Kaźmierczak, Ekin Köken, Piotr Karwowski, Michał Wolny, Tomasz Stępień

**Affiliations:** 1 Department of Mining, Faculty of Geoengineering, Mining and Geology, Wroclaw University of Science and Technology, Wybrzeże Wyspiańskiego 27, 50-370 Wrocław, Poland; 2 Nanotechnology Engineering Department, Engineering Faculty, Abdullah Gul University, Kayseri 38100, Turkey

**Keywords:** crushing technologies, jaw and cone crushers, crushed aggregate, basalt, geometric properties of aggregates

## Abstract

The use of mineral aggregates is related to the increasing demand in construction, railway and road infrastructures. However, mineral aggregates can appear to be of variable quality, directly affecting their suitability for respective earthwork applications. Since the production of mineral aggregates should ensure the standardized, high-quality requirements of the final product, rock-crushing mechanisms should be investigated in a detailed manner. In this context, the aim of the present study is to evaluate and analyze the geometric parameters of basalt aggregates as a result of several rock comminution processes. Basalt aggregates from two deposits in Poland were used in the study. The samples are differentiated regarding both lithological variances, mineral composition as well as the host rock’s tuff content. The rock comminution processes were conducted using two types of crushers, namely the laboratory-scale jaw and cone crushers. The feed for crushing was designed based on the original geometric grain composition and the separated feed in the form of flaky and non-flaky particles. The crushability test results demonstrated that the interparticle compression in the jaw crusher resulted in finer products compared to the one in the cone crusher. It was also observed that the flakiness and shape indexes decreased after crushing, both in the feed with the original geometric composition of the grains and those with flaky and non-flaky particles. Nevertheless, a higher flakiness index was obtained after the crushing of non-flaky particles and a lower one after the crushing of flaky particles. The flakiness index for grains below 16 mm after the crushing process was less than 10%, which indicates a more favorable result compared to the original feed. In addition, it was shown that flaky and non-cubical particles were accumulated in the finest (below 8 mm) and coarsest (above 20 mm) fractions in jaw and cone crushing processes, receiving flakiness and shape indexes ranging up to 80–100%. Finally, it was also observed that the lithological variances of the feed material have a significant impact on the particle size distribution of the product. More profoundly, basalt aggregates with a higher tuff content and weathering degree have a higher degree of crushing. The present study, in this context, provides accurate and satisfying information on understanding the crushing mechanisms of two important crushing equipment as well as their rock-crusher interactions.

## 1. Introduction

One of the common raw materials, which are used in the building and construction industry, is crushed stone or rock aggregate. The increasing demand for crushed stones is related to the growing development of the construction, railway and road infrastructures. Crushed stone is produced by mechanical processes, where several crushing equipments are utilized in crushing–screening plants [1,2,3,4,5,6,7]. Crushed stones should be distinguished by their highest quality, which should be investigated more profoundly from the point of their roughness, angularity, flakiness, particle size distribution, wear and fragmentation resistance [8,9]. In addition, Czinder and Török [10] and Köken and Başpınar Tuncay [11] pointed out that the quality of rock aggregates has a direct relationship to their proper usability and long-term durability.

From engineering geological aspects, the quality of rock aggregates mainly depends on the structure, texture and mineralogical composition of the host rock [9,12,13,14,15]. Moreover, the technical specifications of several aggregate-related engineering structures (i.e., concrete, paving mixtures and railway ballast material) emphasize and declare technical rock properties such as water susceptibility, freezing-thawing resistance, crushing and fragmentation resistance [16,17,18]. From this perspective, Šernas et al. [8] noted that the physical and mechanical properties of rock aggregates are the main factors in choosing the type of rock origin suitable for use in various engineering fields. Therefore, it is logical to suppose that various rock origins, like basaltic rocks, seem to be suitable for their use in concrete and paving mixtures [19,20,21,22].

The physical, mechanical and geometric properties of rock aggregates define the quality of aggregate-related engineering structures. One of the most important aggregate properties is product flakiness. This crucial aggregate property has been investigated from many aspects. For instance, Räisänen and Mertamo [23], Hofer et al. [24], Gawenda [2,25], Rajan and Singh [26,27], Köken [28], Köken and Lawal [29], Strzałkowski et al. [15] and Gawenda et al. [30,31] indicated that the shape properties of rock aggregates are dependent upon the rock manufacturing processes. In other words, different rock crushers have different impacts on rock aggregate flakiness, which ultimately affects the mechanical parameters. More profoundly, Gawenda [2,25] and Gawenda et al. [30] noted that multistage crushing results in a lower content of non-shaped grains. Similarly, Rajan and Singh [26] observed that the aggregate obtained from two-stage crushing had higher grain irregularity compared to four-stage crushing. Strzałkowski et al. [15] also indicated that flaky and non-cubical grains are concentrated in the finest grain fractions.

Apart from these, the origin of feeding material and operational features of the crushing equipment have several impacts on product flakiness. For instance, Eloranta [32] concluded that the particle size distribution (PSD) of feeding material and the closed-side setting (CSS) of cone crushers play a significant role in product flakiness. Bengtsson and Evertsson [33] also studied the factors influencing product flakiness for cone crushers, and they concluded that operational features of the cone crusher (i.e., eccentric speed and CSS) influence product flakiness. Similar results were also obtained by Gang et al. [34]. For other crusher types, such as vertical shaft impact crushers (VSI), the crushing mechanism of VSI affects the shape of fine aggregates in a size range of 3 μm to 250 μm [35]. On the other hand, for the jaw-crushing process, the feeding quantity is associated with crushing energy consumption and product flakiness [29]. Rajan and Singh [36,37] also focused on the variations in rock aggregate morphology produced from various crusher circuits. It was concluded that the rock aggregate angularity is associated with the order of crushers, where the rocks are charged. Recently, rock aggregate flakiness was also investigated by Umashankar et al. [38] using different crushing circuits. Their results indicated that rock aggregates produced from jaw-cone crushers indicated better aggregate shape properties than jaw–jaw crusher association.

Last but not least, Diógenes et al. [39] noted that the jaw crusher produced coarse aggregates with a better combination of sphericity and angularity during the first crushing stage. In contrast, the fine aggregates produced by cone crushers in the final crushing stages were more elongated, but the products had a greater angularity than fine aggregates produced by the primary jaw crusher. 

The fragmentation of an aggregate particle occurs in such ways that depend not only on the intensity and distribution of external forces but also on its internal structure and existing micro-fissures. Problems associated with undesirable shape properties can be solved or minimized by selecting a suitable crusher with a concurrent consideration of rock type [28,29,40,41].

Another aspect discussed in the literature related to the geometric properties of aggregates is the method of determining the shaping qualities of mineral aggregates. 

For the last decades, modern methods based on imaging techniques have been used to determine the shape properties of rock aggregates [42]. These techniques are typically concentrated on the measurement of different particle dimensions based on computer-aided analysis results [43]. In other words, the image analysis of mineral aggregates reduces time-consuming laboratory testing. However, it can have some drawbacks based on the size of mineral aggregates. Persson [43] pointed out that the image analyses of rock aggregates are particularly practicable for fine aggregates because the use of traditional testing methods that measure the size and shape of the fine grains does not seem to be effective. In some cases, the imaging method seems to be ineffective due to the adoption of unnatural grain shapes, which can affect the geometric results [44]. Notwithstanding that image analyzing techniques are a new way of determining the size and shape properties of mineral aggregates, the traditional testing methods to determine the shape properties of rock aggregates seem to be more effective for coarse aggregates because they provide more precise data [39]. Because of that reason, traditional methods based on applied standards have been adopted to determine the size and shape properties of rock aggregates in this study.

The above-mentioned studies put forward a solid basis for the factors influencing product flakiness and the method of determining aggregate grain shape. From a crushing-screening plant design aspect, the main stages of mechanical processing are crushing and sorting (classification). Although these processes are interrelated, it seems that the crushing stage is the key phase influencing the shape properties of the granular materials. The crushing of aggregates is conducted using different crushing equipment, which is distinguished by different technologies and methods of crushing the rocks, with the consequence of varying the quality of the products obtained from this process. The results of the studies reported in the literature were based on originally sampled test material from mines with a specific content of irregular aggregate grains. In order to understand the effect of crushing technology on the geometric parameters of aggregates, it is necessary to investigate the effects of the crushing process with variable content of flaky aggregate grains. In addition, the traditional approach of determining the shape parameters of the aggregate, which is not present in other scientific studies related to rock comminution, provides practical and understandable test results. Therefore, a detailed analysis of the geometric properties of crushed aggregates with reference to the crushing technology is still necessary for the aspect of the production of rock aggregates, which is the purpose of this research article. The considerations presented in this article are a preliminary result of describing the influence of crushing circuits on product flakiness. So, the present study demonstrates detailed analysis results of the shape properties of basaltic rocks from Poland under the influence of different crushing equipment. However, additional laboratory tests should also be helpful in selecting the proper crushing methodology and minimizing the flaky particles in the final product for other rock types.

## 2. Materials and Methods

### 2.1. Materials

The materials for the laboratory tests consisted of high-quality samples from two basaltic rocks from Poland. In this study, 2 averaged samples of crushed aggregates with a grain size of 0–31.5 mm were sampled, each weighing approximately 120 kg. More profoundly, two types of basalt aggregates from the southwestern Poland region were investigated in this study. Note that basalt 1 from the Lubań area petrographically represents a Cenozoic igneous eruptive rock formed from nepheline and tephrite lavas, with an aphanitic texture, which constitutes more than 80% of the host rock around the study area. The texture of basalt 1 is interspersed with phenocrysts and microphenocrysts of olivine, clinopyroxene and magnetite. 

Basalt 2 was sampled from a deposit in the Złotoryja area, which is located approximately 50 km in a straight line from the basalt 1 deposit. 

As with the first sample (basalt 1), its aphanitic background is interspersed with phenocrysts of pyroxene and olivine, and in places, there are air bubbles filled with secondary carbonate minerals. 

**Figure 1:**
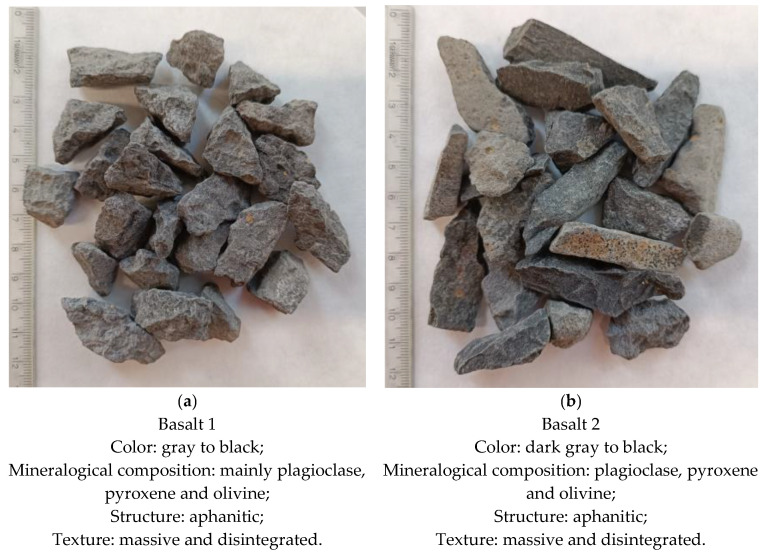
Illustration of two basaltic samples ((**a**) basalt 1; (**b**) basalt 2) tested in this study.

Basalt 2 was characterized by a higher content of tuffs rocks compared to basalt 1. Basalt 1 had a tuff content of less than 5%, while basalt 2 had a tuff content of about 15%. Volcanic tuffs are characterized by lower fragmentation and wear resistance; hence, a rock material with a higher content of tuffs and basalt weathering will have lower strength parameters after the crushing process. Figure 1 illustrates the material tested.

Table 1 shows the physical and mechanical properties of the tested basalt aggregates. The two basalt samples tested have similar properties.

### 2.2. Methods

In the first stage of the laboratory studies, detailed sieve analyses were conducted using the prepared feeding materials. The determination of the grain size distribution was performed in accordance with EN 933-1 [45]. Sieve analyses consisted of separating the materials using square apertured sieves such as 0.063, 0.125, 0.25, 0.5, 1.0, 2.0, 4.0, 5.6, 6.3, 8.0, 10.0, 11.2, 12.5, 14.0, 16.0, 20.0, 22.4, 31.5 mm.

Shape and flakiness indexes were then determined using each of the grain classes above 4 mm. These indices were calculated based on the standards of EN 933-3 [46] and EN 933-4 [47], respectively.

The determination of flakiness index (based on EN 933-3 [46]) consisted of two classification stages. In the first stage, the samples were sieved using square mesh sieve sizes such as 40.0; 31.5; 25.0; 20.0; 16.0; 12.5; 10.0; 8.0; 6.3; 5.0; 4.0 mm, resulting in classified material. In the second stage, the classified materials were sieved using bar sieves with a slot width of D/2.

The flakiness index for the individual size fractions was calculated as the weight of the grains passing through the bar sieves, expressed as a percentage relative to the weight of the grain classes above respective sieves. As a result of this separation, the feeding material is divided into flaky and non-flaky particles. The flakiness index indicates the percentage of flaky grains in relation to the weight of the feed material. The flakiness index is determined by adopting specific size fractions.

The shape index of aggregate particles (based on EN 933-4 [47]) is also identified based on the ratio between the maximum dimension of the grain defined by the greatest distance separating two parallel planes tangential to the particle surface and the thickness of the minimum grain dimension. It is determined using a typical Schultz caliper. The separation process ended up with cubical and non-cubical particles in the feeding material.

In the next stage, the feeding materials were averaged and prepared for crushability tests. Based on the above experimental procedure, approximately 40 kg of each basaltic sample ranging from 16 to 31.5 mm were prepared.

Studies on the efficiency of the crushing process of basalt aggregates were conducted using two types of laboratory-scale rock crushers. They were jaw and cone crushers. In the context of the crushability tests, the gap width of both crushers was set at 18 ± 1 mm.

**Figure 2:**
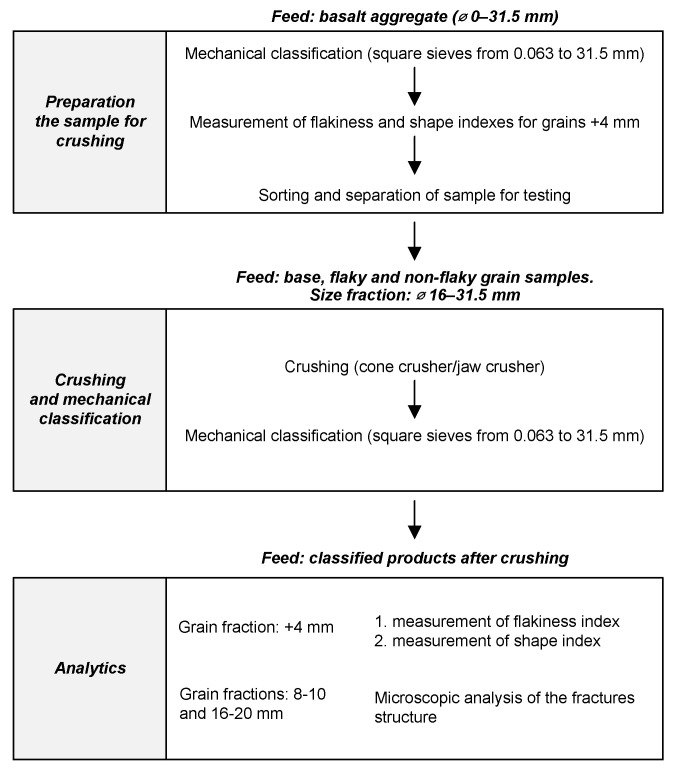
Experimental flowchart of the crushability tests.

The prepared base samples and averaged flaky and non-flaky grain samples were crushed using laboratory-scale rock crushers. A total of six different crushing operations were conducted using prepared samples composed of basalt 1 and basalt 2. The products obtained from crushing were analyzed granulometrically, separating individual grain classes, and then the shape and flakiness indexes were determined for each grain class. In addition, the dust content—grains below 0.063 mm—was also determined for each sample before and after crushing. The experimental flowchart for the crushability tests is given in Figure 2.

For each crushed product, detailed structural analyses of the cracks formed by crushability tests were also performed. It is important to note that revealing the characteristics of cracks formed by different crushers gives practical information on the quality of aggregates under the domination of repeated surcharge loads. 

Micro-crack structure studies were conducted using a Motic SMZ-168 Series microscope, Nikon Digital Sight DS-Fi2 camera and Nikon NIS-Elements Basic Research software. The micro-crack studies were conducted using the products with 8–10 mm and 16–20 mm grain size classes. 

**Figure 3:**
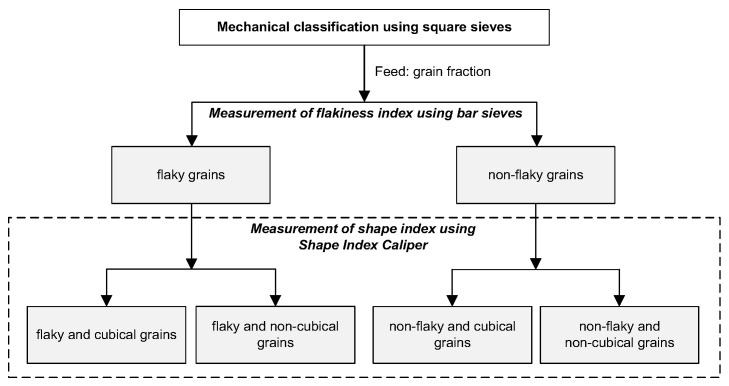
Grain classification scheme of samples before and after crushing.

In the literature, the terms irregular and regular grains are very often used to refer to the non-flaky and flaky grains [15]. According to the corresponding author’s knowledge, it is not a correct interpretation. Flaky grains are not necessarily non-cubical and conversely, so distinguishing between these four terms is very important in the classification of crushed aggregate grains. In this study, a novel methodology with proper identifications was adopted to separate grain geometries in terms of flakiness index into flaky and non-flaky grains. Then, in terms of the shape index, another two classes were separated from these two subgroups by means of the Schultz caliper, considering the shape of the grains. Thus, from each sample after crushing, the following grains were separated into grain classes: flaky cubical grains, flaky non-cubical grains, non-flaky cubical grains and non-flaky non-cubical grains, respectively. The grain classification scheme of the pre- and post-crushing samples is shown in Figure 3. 

The research presented in this paper represents a series of standard operations performed during the testing of crushed aggregates according to existing and applicable standards. The combination of these operations into a unique testing scheme, especially in terms of the separate crushing of flaky and non-flaky grains, constitutes a new testing methodology based on standardized testing equipment.

## 3. Results and Discussion

**Figure 4:**
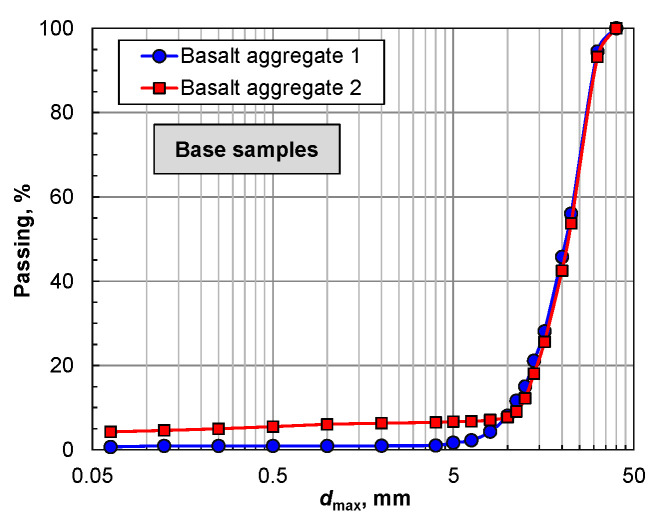
Grain classification scheme of samples before and after crushing.

Basalt aggregate samples were first subjected to granulometric analysis. The grain size distribution is determined by mechanical classification, which aims to divide the aggregate sample into several size fractions, each of which consists of particles within defined size limits. Figure 4 shows the results of the grain size distribution analysis for the primary samples of the tested aggregates. The basalt aggregates are characterized by similar grain sizes, except for the finest grain classes. Basalt 2 was characterized by a significantly higher content of finer grain classes than basalt 1. The values on the horizontal axis are presented on a logarithmic scale in accordance with the requirements of EN 933-1 [45].

**Figure 5:**
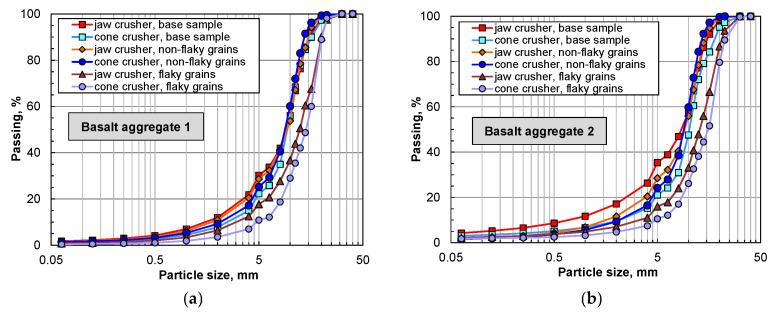
Particle size distribution of aggregates after crushing: (**a**) basalt 1; (**b**) basalt 2.

Figure 5 shows the grain composition of the samples after crushing in the two types of crushers with reference to several grain shapes. From the analysis of the grain size distribution curves, it can be noted that the crushing of flaky grains results in a noticeably finer grain size of the post-crushing product compared to the crushing of the original sample as well as the non-flaky grains. For both samples, despite the same adopted outlet gap width of the crushers, the products after crushing in the jaw crusher had a finer grain size than the products after crushing in the cone crusher. This is the result of interparticle compression in jaw crushing [29].

After analyzing the grain size distribution and determining the shape and flakiness indexes, the basalt aggregate samples were crushed in the jaw crusher and the cone crusher. For the analysis of the crushing process, the original sample should be perceived as the aggregate sampled directly in its original state in the processing system. The feeds in the form of flaky and non-flaky grains, on the other hand, were separated before the crushing process from the original sample and crushed during separate crushing operations. 

It was observed that for the rock aggregates with a high content of tuffs and consisting of several weathering signs such as sericitization and argillization, the degree of crushing between the original sample feed and the flaky grain feed was higher than that of the sample with a low content of grains other than pure basalt. 

This finding is attributed to the fact that the variable lithological composition of the feed in the deposit will significantly determine the grain size distribution of the product after crushing, regardless of the type of crusher used for crushing.

Table 2 shows the geometric indexes for the original samples before crushing. For coarse grain classes above 20 mm, basalt 1 was characterized by lower shape and flakiness index values. The finer the grain class, in this case, basalt 1, the higher the content of non-cubical and flaky grains relative to basalt 2. It was also observed that in the case of the fine grain class of basalt 2, the grains of tuffs and basalt weathering are characterized by a cubic shape, and the edges of the grains are clearly rounded.

**Figure 6:**
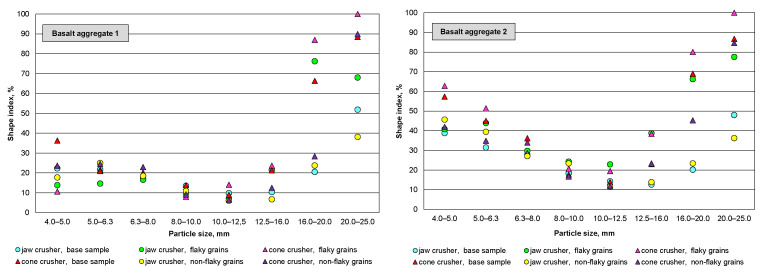

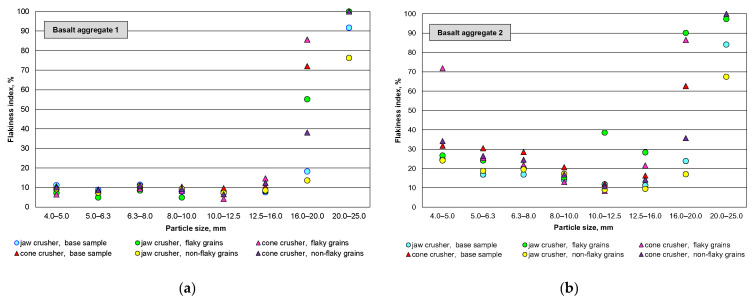
Comparison of geometric indexes for analyzed basalt aggregates: (**a**) basalt 1; (**b**) basalt 2.

Table 3 and Table 4 and Figure 6 show the geometric structure of the mineral grains after crushing in both types of crushers, with a division by the type of feed that was directed to the crushing process. Lower flakiness and shape indexes were achieved after the crushing process, both in the feed with the original geometric grain composition and in the feed with flaky and non-flaky grains. Overall, the flakiness index of grains below 16 mm after the crushing process was below 10%, which indicates a more favorable result in relation to the geometric parameters of the feed. Despite the generally lower flakiness and shape indexes after the crushing process, it was noted that a higher flakiness index is achieved when crushing non-flaky grains and a lower one after crushing flaky grains. The reason for this phenomenon may be interpreted just as the easier destruction of the grain structure, which is weakened at the point of the smallest grain size. The concentration of flaky and non-flaky grains, after crushing, was in the finest (less than 8 mm) and coarsest (greater than 20 mm) size fractions, which were determined to be between 80–100%. Regardless of the geometrical parameters of the feed, whether it was the original sample or samples separated due to grain flakiness, basalt 2 had higher shape and flakiness index values after each crushing attempt compared to basalt 1. The flakiness index for grain classes below 16 mm was below 10%, showing an improvement in this parameter compared to the feed (Table 2). Gawenda et al. [30] propose that the geometric parameters of aggregates are modified as a result of the secondary classification of the products after the crushing process. This classification could be conducted on slot screens so as to separate out flaky grains. Flaky grains, in this case, would constitute a rejection. Our analyses indicate that improving the parameters of the flaky grains can be obtained by re-crushing them. 

For both basalt samples, rock crushing in a cone crusher system resulted in a higher content of non-cubical and flaky grains (especially for classes above 16 mm) in the product after crushing, relative to crushing in a jaw crusher system, regardless of the geometric composition of the feed grains. This is consistent with the results presented by Diógenes et al. [39]. Similarly, better product performance after crushing was obtained for single-stage crushing in a jaw crusher. Therefore, it is recommended that a suitable rock-crushing mechanism should be conducted using a suitable combination of different rock crushers [48].

**Figure 7:**
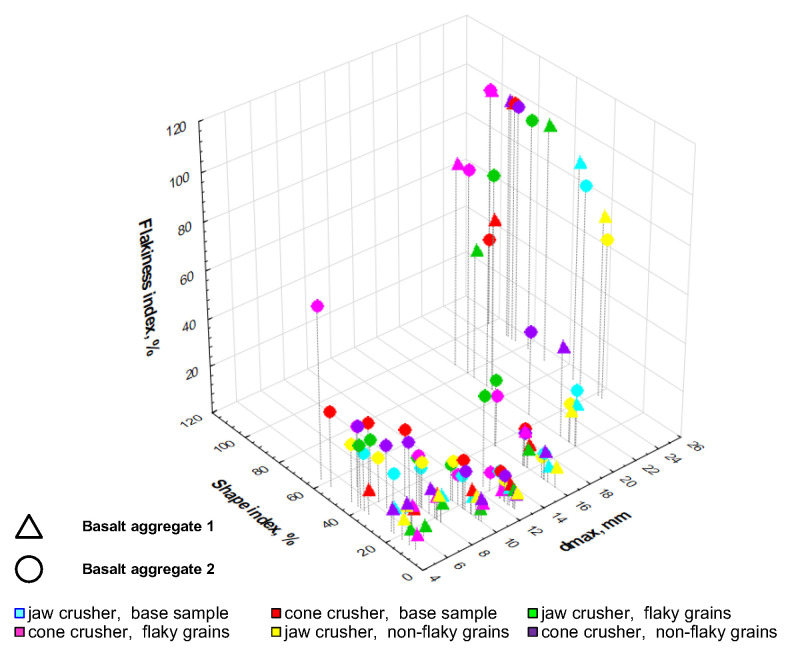
Three-dimensional distribution characteristics of geometric parameters in grain classes.

Figure 7 shows the geometrical characteristics of the basalt aggregates after jaw and cone crushing. The graph was presented based on a three-parameter system, determining the distribution of shape and flakiness indexes for each grain class. This allowed a three-parameter analysis of the distribution of geometric properties of the products obtained after crushing. The colors on the graph mark the type of feed and the type of crusher used for crushing, while the shape of the marker indicates the deposit from which the material comes.

Based on the analysis of the three-dimensional distribution of the geometrical properties of the analyzed aggregates (Figure 7), it can be concluded that basalt 2 had a higher content of flaky and non-cubical grains, especially in the fine grain class system. The basalt aggregate with a low degree of weathering and low tuff content is generally crushed in the form of non-flaky cubical grains. Aggregates with lower strength parameters, as in the case of the aggregates studied—basalts with a higher content of grains other than pure basalt, show worse geometrical parameters after crushing, as confirmed by Gawenda et al. [49].

**Figure 8:**
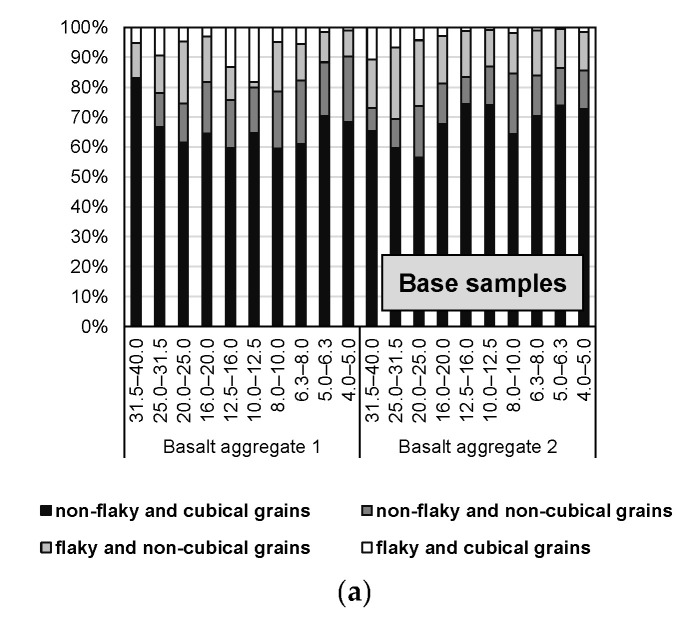

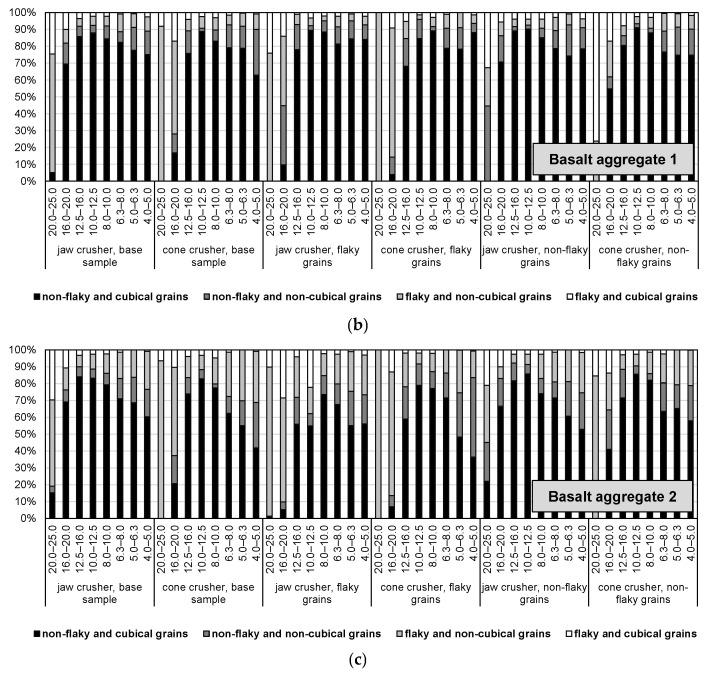
Characteristics of the distribution of grains with specific geometrical parameters in grain classes: (**a**) base samples; (**b**) basalt 1; (**c**) basalt 2.

Figure 8 also shows the percentage characteristics of the mineral grains classified according to Figure 3 in the before- and after-crushing samples, with the percentage division into flaky cubical grains, flaky non-cubical grains, non-flaky cubical grains and non-flaky non-cubical grains. Each grain class was operationally summed to 100%, regardless of its mass yield.

From the grain size distribution analysis, the rock crushing of basalt 1 in the product after crushing increased the proportion of non-flaky and cubical grains compared to the original sample (except for the coarsest grain class) from about 60 to about 80%, which was associated with a general decrease in the content of especially non-flaky non-cubical grains. The change in the percentage structure of the grain distribution after crushing for aggregate sample 2, with its higher tuff and weathering content, is quite different. Namely, a clear increase in the content of flaky grains, in general, was noticed in the samples after crushing, irrespective of the type of crusher and feed structure.

On microscopic analysis of the original samples, the coarse grain classes were observed to be dominated by grains of pure basalt with single grains of secondary crystallized olivine in the aphanitic body, according to data from Strzałkowski et al. [15]. In both basalt aggregates, the single cracks occurring in the coarser grain classes are mainly in the pattern of secondary olivine crystallization. In addition, single cracks are visible in the basalt aggregate 2, which are rather related to the weathering of the material and are original cracks unrelated to the crushing system of these aggregates.

**Figure 9:**
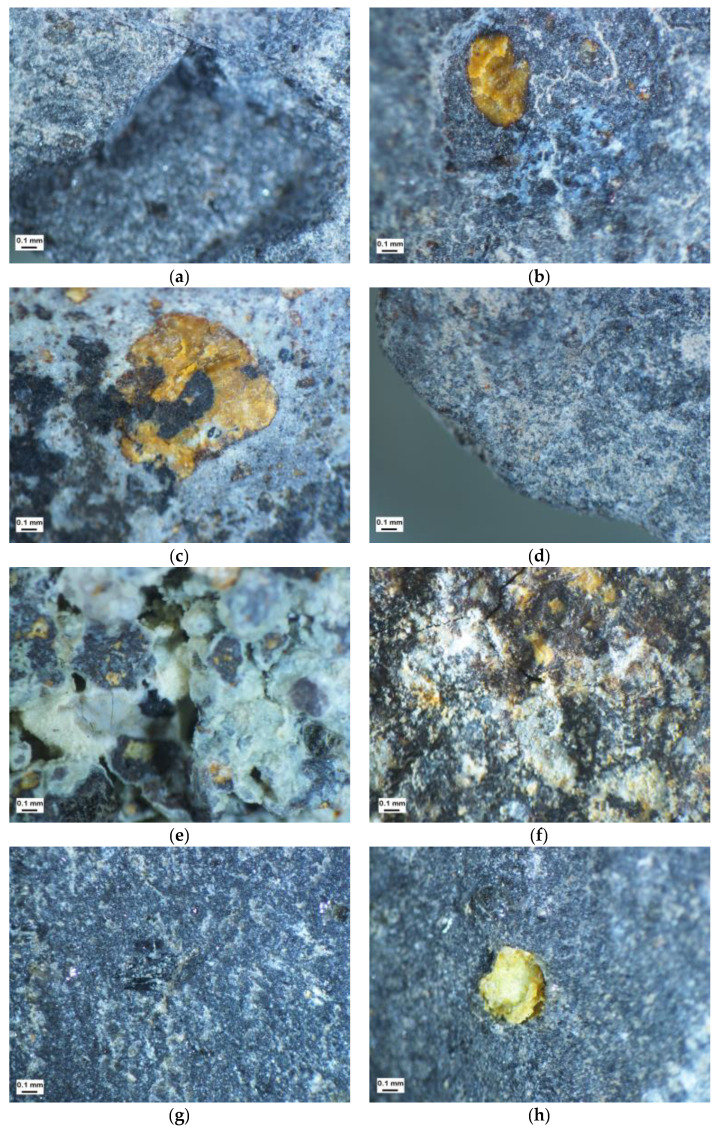
Characteristics of cracks in original samples: (**a**) Basalt aggregate 1, non-flaky grains, +8 mm; (**b**) Basalt aggregate 1, non-flaky grains, +16 mm; (**c**) Basalt aggregate 1, flaky grains, +8 mm; (**d**) Basalt aggregate 1, flaky grains, +16 mm; (**e**) Basalt aggregate 2, non-flaky grains, +8 mm; (**f**) Basalt aggregate 2, non-flaky grains, +16 mm; (**g**) Basalt aggregate 2, flaky grains, +8 mm; (**h**) Basalt aggregate 2, flaky grains, +16 mm.

For the microscopic characterization of the fine grains, it was noted that, as in the coarser grain classes, the cracks form concentric networks from secondary crystallization, with additional cracks in the fine classes from the edges of the grains towards their center of gravity, especially in the flaky grain structure system (Figure 9). Fine grains of weathered basaltic and tuffs were characterized by distinctly rounded edges with a less developed edge line. The more cubic grains lacked major depressions and jagged structures.

**Figure A1:**
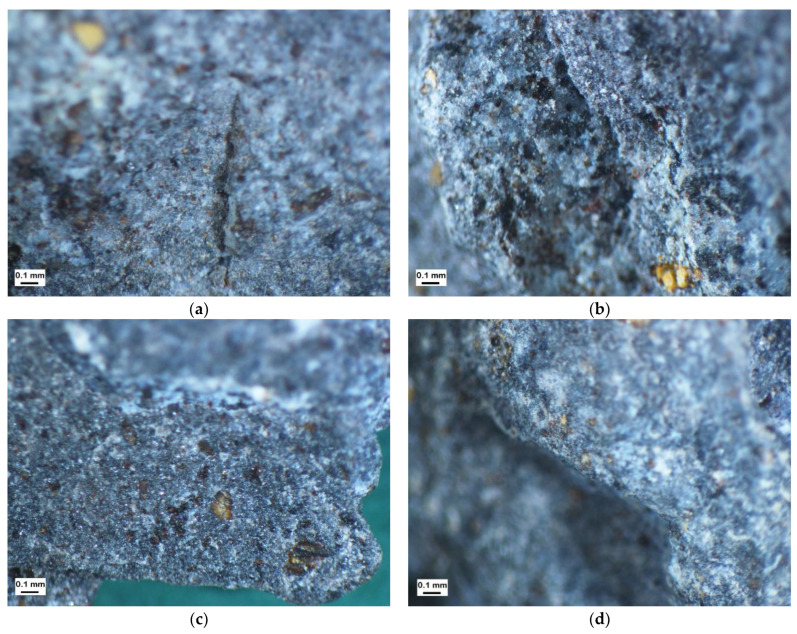
Characteristics of cracks in samples after crushing in jaw crusher (feed—original sample): (**a**) Basalt aggregate 1, non-flaky grains, +8 mm; (**b**) Basalt aggregate 1, non-flaky grains, +16 mm; (**c**) Basalt aggregate 1, flaky grains, +8 mm; (**d**) Basalt aggregate 1, flaky grains, +16 mm.

**Figure A2:**
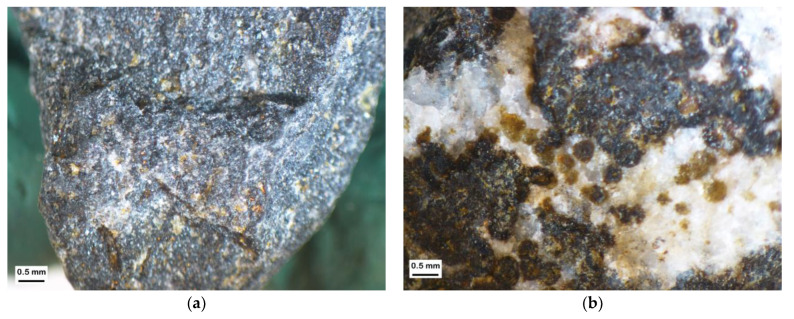

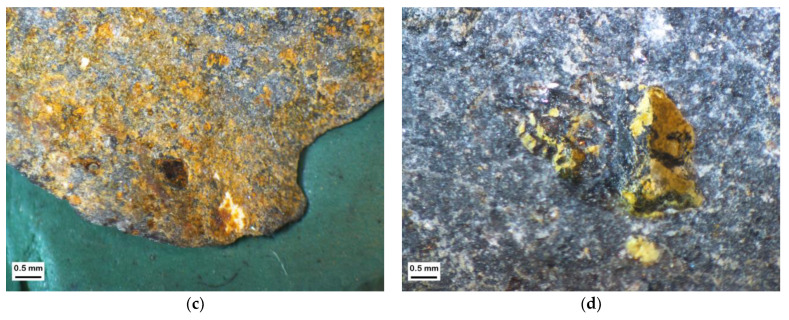
Characteristics of cracks in samples after crushing in jaw crusher (feed—original sample): (**a**) Basalt aggregate 2, non-flaky grains, +8 mm; (**b**) Basalt aggregate 2, non-flaky grains, +16 mm; (**c**) Basalt aggregate 2, flaky grains, +8 mm; (**d**) Basalt aggregate 2, flaky grains, +16 mm.

**Figure A3:**
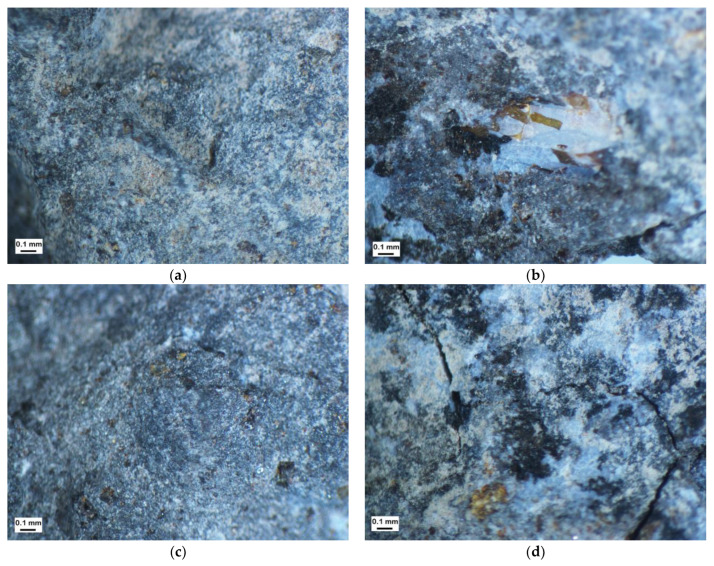
Characteristics of cracks in samples after crushing in cone crusher (feed—original sample): (**a**) Basalt aggregate 1, non-flaky grains, +8 mm; (**b**) Basalt aggregate 1, non-flaky grains, +16 mm; (**c**) Basalt aggregate 1, flaky grains, +8 mm; (**d**) Basalt aggregate 1, flaky grains, +16 mm.

**Figure A4:**
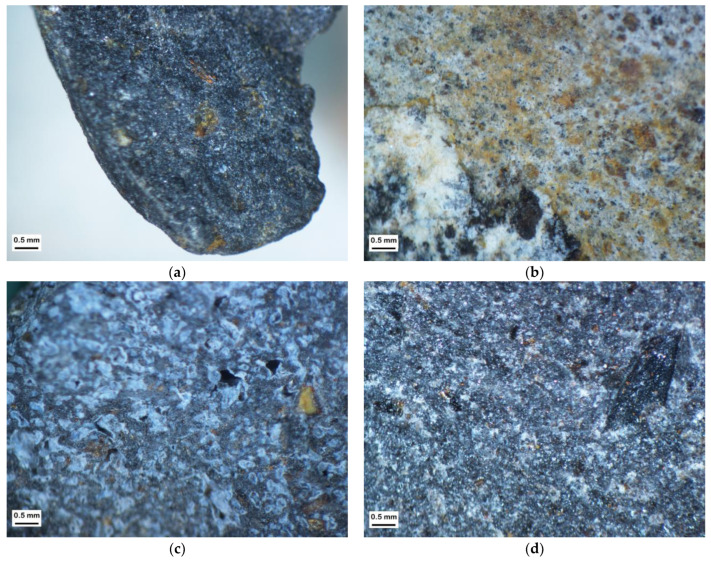
Characteristics of cracks in samples after crushing in cone crusher (feed—original sample): (**a**) Basalt aggregate 2, non-flaky grains, +8 mm; (**b**) Basalt aggregate 2, non-flaky grains, +16 mm; (**c**) Basalt aggregate 2, flaky grains, +8 mm; (**d**) Basalt aggregate 2, flaky grains, +16 mm.

**Figure A5:**
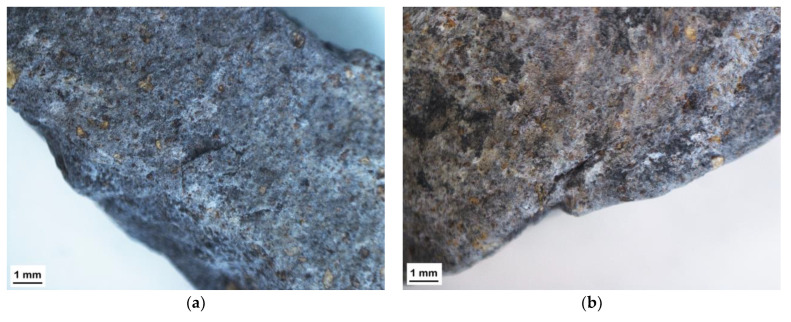

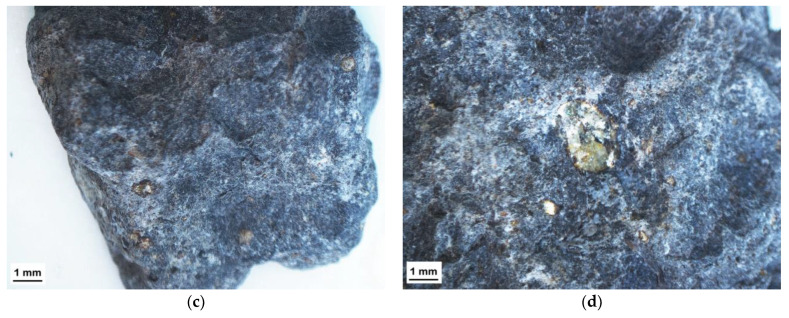
Characteristics of cracks in samples after crushing in jaw crusher (feed—flaky grains): (**a**) Basalt aggregate 1, non-flaky grains, +8 mm; (**b**) Basalt aggregate 1, non-flaky grains, +16 mm; (**c**) Basalt aggregate 1, flaky grains, +8 mm; (**d**) Basalt aggregate 1, flaky grains, +16 mm.

**Figure A6:**
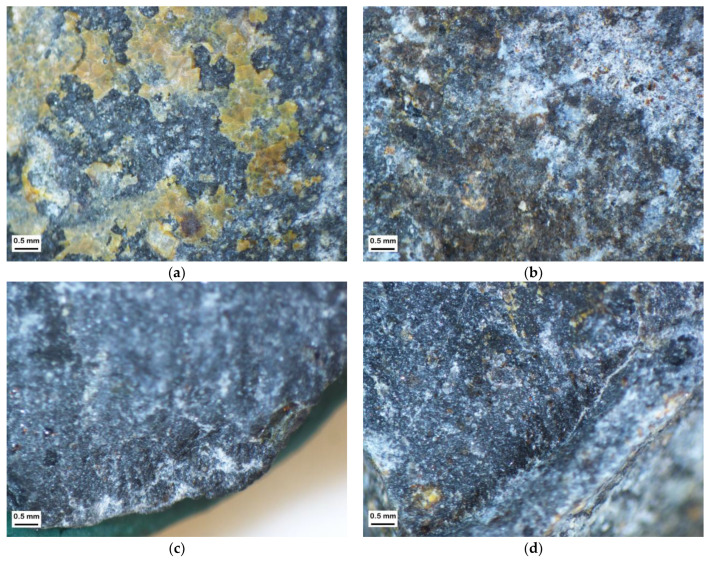
Characteristics of cracks in samples after crushing in jaw crusher (feed—flaky grains): (**a**) Basalt aggregate 2, non-flaky grains, +8 mm; (**b**) Basalt aggregate 2, non-flaky grains, +16 mm; (**c**) Basalt aggregate 2, flaky grains, +8 mm; (**d**) Basalt aggregate 2, flaky grains, +16 mm.

**Figure A7:**
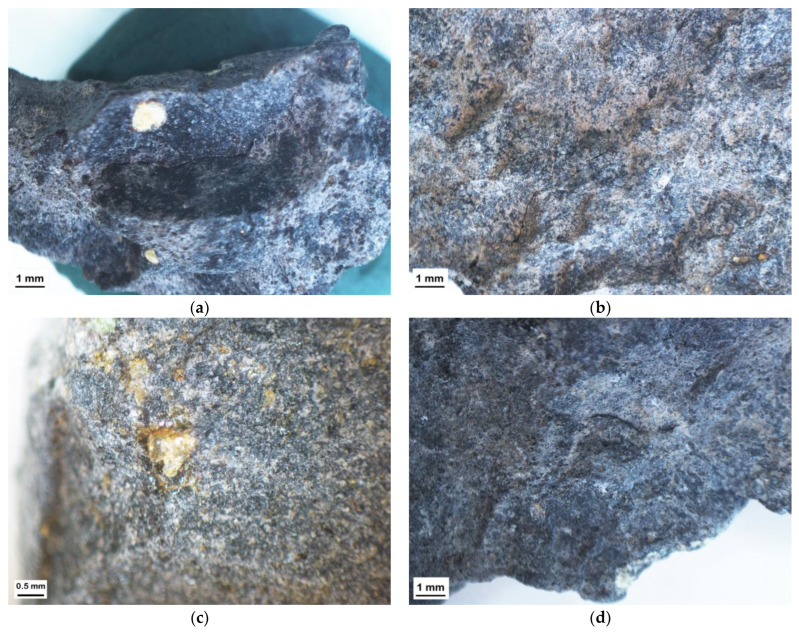
Characteristics of cracks in samples after crushing in cone crusher (feed—flaky grains): (**a**) Basalt aggregate 1, non-flaky grains, +8 mm; (**b**) Basalt aggregate 1, non-flaky grains, +16 mm; (**c**) Basalt aggregate 1, flaky grains, +8 mm; (**d**) Basalt aggregate 1, flaky grains, +16 mm.

**Figure A8:**
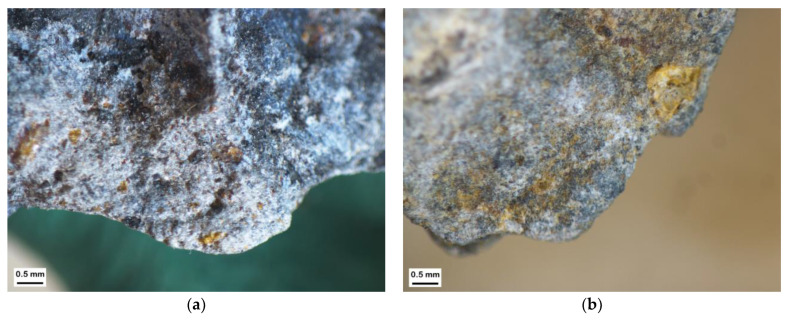

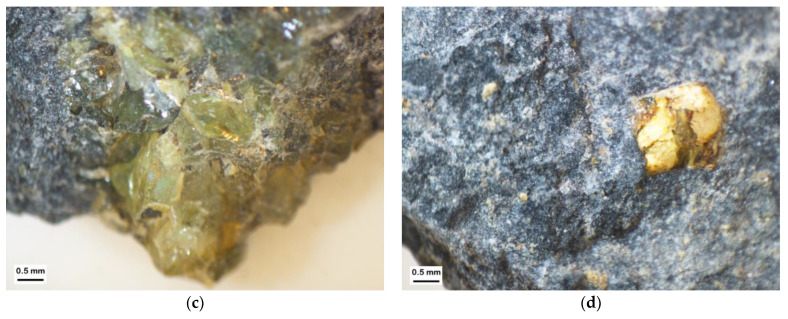
Characteristics of cracks in samples after crushing in cone crusher (feed—flaky grains): (**a**) Basalt aggregate 2, non-flaky grains, +8 mm; (**b**) Basalt aggregate 2, non-flaky grains, +16 mm; (**c**) Basalt aggregate 2, flaky grains, +8 mm; (**d**) Basalt aggregate 2, flaky grains, +16 mm.

**Figure A9:**
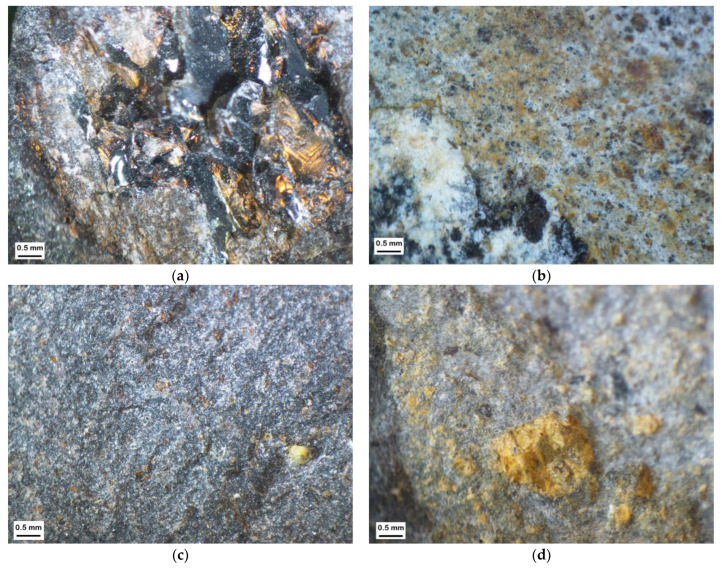
Characteristics of cracks in samples after crushing in jaw crusher (feed—non-flaky grains): (**a**) Basalt aggregate 1, non-flaky grains, +8 mm; (**b**) Basalt aggregate 1, non-flaky grains, +16 mm; (**c**) Basalt aggregate 1, flaky grains, +8 mm; (**d**) Basalt aggregate 1, flaky grains, +16 mm.

**Figure A10:**
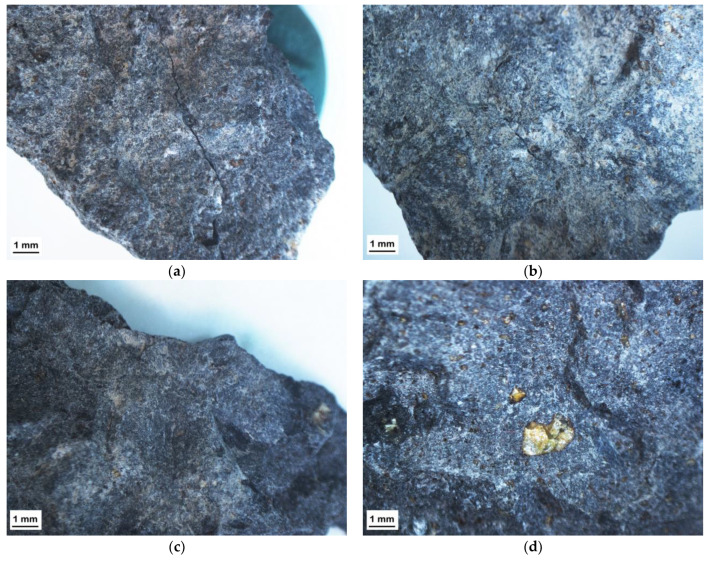
Characteristics of cracks in samples after crushing in jaw crusher (feed—non-flaky grains): (**a**) Basalt aggregate 2, non-flaky grains, +8 mm; (**b**) Basalt aggregate 2, non-flaky grains, +16 mm; (**c**) Basalt aggregate 2, flaky grains, +8 mm; (**d**) Basalt aggregate 2, flaky grains, +16 mm.

**Figure A11:**
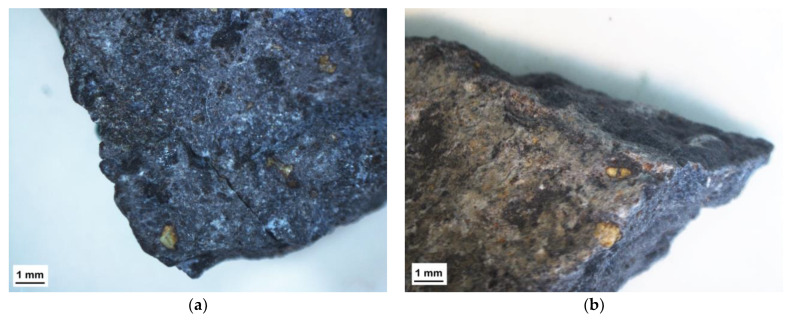

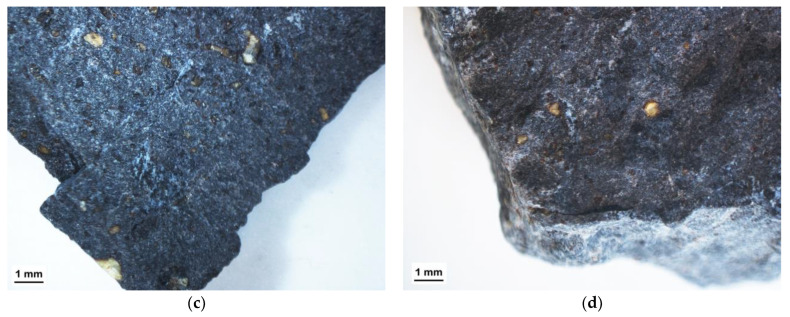
Characteristics of cracks in samples after crushing in cone crusher (feed—non-flaky grains): (**a**) Basalt aggregate 1, non-flaky grains, +8 mm; (**b**) Basalt aggregate 1, non-flaky grains, +16 mm; (**c**) Basalt aggregate 1, flaky grains, +8 mm; (**d**) Basalt aggregate 1, flaky grains, +16 mm.

**Figure A12:**
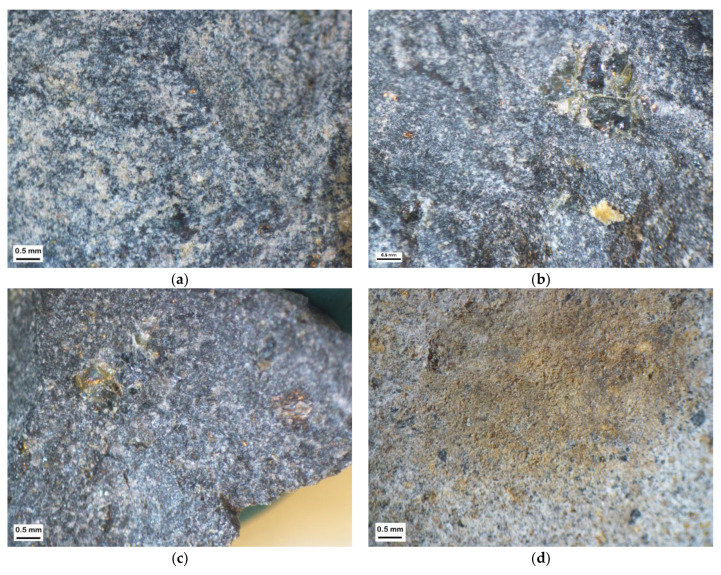
Characteristics of cracks in samples after crushing in cone crusher (feed—non-flaky grains): (**a**) Basalt aggregate 2, non-flaky grains, +8 mm; (**b**) Basalt aggregate 2, non-flaky grains, +16 mm; (**c**) Basalt aggregate 2, flaky grains, +8 mm; (**d**) Basalt aggregate 2, flaky grains, +16 mm.

The crack characteristics of the samples after crushing in the form of photographs from the microscopic analysis are included in Appendix A (Figure A1, Figure A2, Figure A3, Figure A4, Figure A5, Figure A6, Figure A7, Figure A8, Figure A9, Figure A10, Figure A11 and Figure A12). On the basis of the analysis, it was identified that for the coarse grain classes for both aggregates studied, the cracks are mainly associated with secondary mineralization grains with fine cracks in the network. Moreover, it was noted that secondary cracks mainly associated with crushing propagate radially from these grains. It has been observed that secondary deeper cracks from the outer edges of these grains occur in flaky grains after crushing, irrespective of the shape of the supernatant grains. In the flaky grains after crushing, the crack structure is clearly larger than in the flaky grains in the original sample.

Independently of this, it was also noted that for both grain classes tested in basalt aggregate 2, the cracks on the surface of the mineral grains after crushing were more frequent than in the mineral grains of aggregate 1. This observation indicates that crushing of geologically heterogeneous feed results in products with worse geometric parameters.

The analysis of the fine grain structure showed that clearly crushed tuff grains pass into the fine grades. The tuffs in this system are generally characterized by a cubical shape. On the other hand, the grains of pure basalt are in the form of flaky grains with sharp edges and a network of individual cracks radiating out from the edges (the grains at this point have the lowest thickness). As with the grade above 16 mm, if crystals were present in the grains in the aphanitic body, the cracks were mainly in their structure.

Previous studies presented in the literature [25,28,29,31,39,40,41,48,49] mainly focused on the analysis of geometric parameters for feed material, which is unseparated material sampled directly from the deposit or from the technological line of the processing plant. In this study, the focus was on the analysis of a system in which the feed material for the subsequent crushing stages was separated into grains with strictly defined geometric parameters: shape and flakiness. The study showed that there are significant differences in the geometric parameters of aggregates obtained by crushing flaky and non-flaky grains. In addition, an analysis of the geometric parameters of the post-crushing products in terms of the geological parameters of the crushed feed showed that the separation of the feed grains into flaky and non-flaky grains can improve the final parameters of the commercial product. This is possible with single-stage crushing. Especially in the case of weathered basalts, jaw crusher systems should be used.

## 4. Conclusions

Rock aggregates should be characterized by their high quality because they must fulfill high requirements depending on their specific use. Based on rock aggregate manufacturing, the most important mechanical processing stages are crushing and screening. However, the crushing stage is a key step that affects the shape properties of the product. Therefore, the analysis of the geometric properties of aggregates in relation to this stage in the crushing technology allows for the appropriate selection of the technology and method of rock crushing and, consequently, the achievement of high-quality aggregates with the parameters desired by customers.

The conducted tests on the crushing of basalt aggregate, using two types of crushers: jaw crusher and cone crusher, showed the following:

- Lower flakiness and shape indexes after the crushing process, both in the feed with the original geometric composition of the grains and in the feed in the form of flaky and non-cubical particles;
- A higher flakiness index after the crushing process for non-cubical particles and a lower one after the crushing of flaky particles;
- Flakiness indexes for grains below 16 mm after the crushing process of less than 10%, which indicates a more favorable result in relation to the feed;
- An accumulation of flaky and non-cubical particles in the finest (below 8 mm) and coarsest (above 20 mm) fractions, with flakiness and shape indexes as high as 80–100%;
- Higher content of flaky and non-cubical particles for grades above 16 mm after crushing in a cone crusher;
- The presence of turf grains and weathered basalt, mainly in the finest grain classes;
- After crushing, higher contents of flaky and non-cubical particles in samples with higher contents of components other than pure basalt than in pure basalt aggregate;
- Relatively more micro-cracks in post-crushing samples of basalt from a deposit with higher tuff content;
- Reduced quality parameters of the aggregates, resulting in a high content of flaky and non-cubical particles in post-crushing samples due to the increase in tuff content in the sample.

The most appropriate crushing method: if the aggregates are to be wear and fragmentation resistant and contain a high content of non-flaky and cubical grains, its crushing system should be based on jaw crusher technology. However, it should be mentioned that during jaw crushing, the phenomenon of interparticle compression should be considered in further studies. Based on these observations, the structure and composition of the rock should be considered when selecting mineral aggregate production technology, apart from the expected geometrical characteristics of the produced aggregates. In order to ensure a proper approach to the choice of rock crushing technology and method, it seems appropriate to conduct further and broader studies in this area with other aggregates of igneous origin and develop an appropriate crushing procedure that includes the detailed lithological composition of the rocks.

## Figures and Tables

**Table 1:** Average physical and mechanical properties values of two basaltic samples.

Properties of Aggregates	Basalt Aggregate 1	Basalt Aggregate 2
Bulk density (ρ), g/cm^3^	3.10	3.03
Absorptivity (WA_24_), %	0.45	0.70
Micro-Deval index (M_DE_), %	8.58	8.82
Los Angeles index (LA), %	9.39	9.45

**Table 2:** Average shape and flakiness indexes for the analyzed igneous aggregates.

Particle Size, mm	Basalt Aggregate 1		Basalt Aggregate 2	
	Shape Index	Flakiness Index	Shape Index	Flakiness Index
31.5–40.0	11.77	16.87	23.94	26.84
25.0–31.5	24.06	21.96	33.59	30.56
20.0–25.0	33.84	25.51	39.15	26.26
16.0–20.0	32.21	18.27	29.56	18.82
12.5–16.0	29.33	24.32	24.37	16.60
10.0–12.5	33.53	20.03	25.10	12.97
8.0–10.0	35.62	21.36	33.68	15.86
6.3–8.0	33.42	18.04	28.51	18.34
5.0–6.3	27.98	12.01	25.62	26.72
4.0–5.0	30.58	9.33	25.69	15.05

**Table 3:** Average shape and flakiness indexes for the analyzed basalt aggregate 1 after crushing.

Particle Size, mm	Crusher Type											
	Jaw Crusher		Cone Crusher		Jaw Crusher		Cone Crusher		Jaw Crusher		Cone Crusher	
	Feed											
	Base Sample				Flaky Grains				Non-Flaky Grains			
	Shape Index	Flakiness Index	Shape Index	Flakiness Index	Shape Index	Flakiness Index	Shape Index	Flakiness Index	Shape Index	Flakiness Index	Shape Index	Flakiness Index
20.0–25.0	51.83	91.72	88.53	100.00	68.02	100.00	100.00	100.00	38.13	76.26	89.89	100.00
16.0–20.0	20.48	18.20	66.30	72.01	76.20	55.13	86.96	85.69	23.72	13.60	28.31	38.11
12.5–16.0	10.35	7.58	21.34	10.50	21.56	8.05	23.63	14.63	6.69	8.54	12.48	12.35
10.0–12.5	9.80	7.39	8.97	9.62	7.21	7.68	14.02	4.15	5.99	7.18	6.48	6.54
8.0–10.0	13.48	7.92	13.87	10.37	9.51	4.87	7.97	8.20	10.94	9.31	9.18	9.35
6.3–8.0	16.84	11.43	19.26	10.63	16.52	8.45	20.16	9.36	18.48	10.74	22.95	11.17
5.0–6.3	21.69	8.70	20.90	8.04	14.63	4.87	21.59	9.05	24.96	7.40	24.53	8.62
4.0–5.0	22.33	11.15	36.29	10.62	13.81	7.23	10.64	6.45	17.72	9.17	23.62	9.96

**Table 4:** Average shape and flakiness indexes for the analyzed basalt aggregate 2 after crushing.

Particle Size, mm	Crusher Type											
	Jaw Crusher		Cone Crusher		Jaw Crusher		Cone Crusher		Jaw Crusher		Cone Crusher	
	Feed											
	Base Sample				Flaky Grains				Non-Flaky Grains			
	Shape Index	Flakiness Index	Shape Index	Flakiness Index	Shape Index	Flakiness Index	Shape Index	Flakiness Index	Shape Index	Flakiness Index	Shape Index	Flakiness Index
20.0–25.0	47.97	84.10	86.69	100.00	77.53	97.31	100.00	100.00	36.19	67.43	84.60	100.00
16.0–20.0	20.18	23.82	68.96	62.66	66.20	90.13	80.05	86.51	23.36	17.02	45.31	35.71
12.5–16.0	12.62	11.23	22.98	16.31	38.80	28.30	38.39	21.54	13.83	9.44	23.33	14.21
10.0–12.5	14.26	11.84	13.84	12.02	22.88	38.52	19.49	8.30	11.57	9.00	11.81	10.76
8.0–10.0	18.29	13.98	17.68	20.68	24.28	15.26	20.75	12.94	23.41	17.23	16.73	16.91
6.3–8.0	27.72	16.87	36.20	28.52	29.75	20.27	28.57	21.77	27.07	19.52	33.99	24.50
5.0–6.3	31.44	16.80	45.02	30.48	43.90	24.07	51.43	25.44	39.43	18.74	34.84	26.44
4.0–5.0	38.80	24.81	57.34	31.74	40.77	26.72	62.79	71.87	45.66	24.12	41.93	34.16

## Data Availability

Not applicable.
